# Results of Maladaptive Behavior Gender Characteristics Diagnostics in Primary School Children with Disabilities (Pilot Study)

**DOI:** 10.1192/j.eurpsy.2022.601

**Published:** 2022-09-01

**Authors:** E. Fadeeva, E. Shakun

**Affiliations:** National Medical Research Centre for Psychiatry and Narcology n.a. V.Serbsky Russian Federation Ministry of Health, Department Of Preventive Care In Narcology, Moscow, Russian Federation

**Keywords:** School-Aged Children, Behavioral Disorders, Maladaptive Behavior

## Abstract

**Introduction:**

The maladaptive children behavior analysis is important for determining effective methods of prevention and care.

**Objectives:**

The aim of the study was to identify general and gender-specific features of maladaptive behavior in children of primary school age with disabilities.

**Methods:**

The sample included 77 children 8.6±1.03 years of age, among them 57 boys and 20 girls. Maladaptive children behavior was assessed using VABS. The statistical significance of the differences between variables was determined by Pearson`s Chi-squared test. Indicators of maladaptive behavior were assessed by calculation of frequency distribution and contingency tables.

**Results:**

Maladaptive behavior features common for both genders included impulsivity, physical aggression, taunting, teasing and bullying, insensitivity to others, having poor eye contact. Having a hard time paying attention was statistically significantly more common among boys (p≤0.05). Boys were more likely to disobey and defy those in authority, to lie, cheat or steal. A specific feature of maladaptive behavior for girls was having eating difficulties and overly dependent behavior on caregivers or siblings. Comparative analysis of the additional VABS section results showed that obsession with thoughts or activities predominated among boys, as well as expression of thoughts that do not make sense. The following indicators were found only in boys: strange habits or ways (makes repetitive noises, odd hand movements, etc.) (14%), bizarre speech (conversations with self in public, repeating the same word or phrase, etc.) (7%).

**Conclusions:**

Described signs of children’s maladjustment can be used for the purposes of diagnostics, prevention and care.

**Disclosure:**

No significant relationships.

